# Supplementary material for: Can we study 3D grid codes non-invasively in the human brain? Methodological considerations and fMRI findings
Source: Neuroimage. 2019 Feb 1;186:667–78. doi: 10.1016/j.neuroimage.2018.11.041 (PMC6347569; doi:10.1016/j.neuroimage.2018.11.041)
Supplement: Supplementary Material_2 [file mmc1.docx]

Supplementary Material

**Can we study 3D grid codes non-invasively in the human brain? Methodological considerations and fMRI findings**

**Misun Kim, Eleanor A. Maguire**

Wellcome Centre for Human Neuroimaging, Institute of Neurology,

University College London, London WC1N 3AR, UK


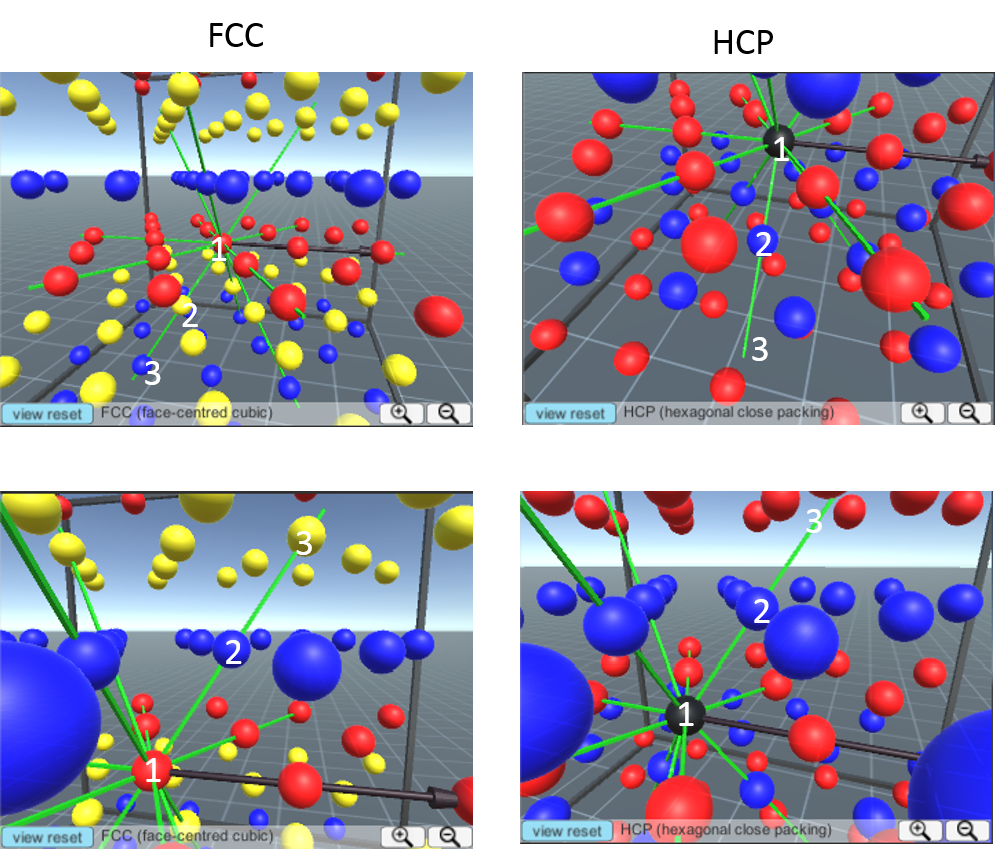


**Supplementary Fig. 1.** Grid axes of the FCC and HCP arrangements.

FCC is a pure lattice with centre-symmetry. If one moves along the main grid axis from one node, every grid field will be passed (three nodes labelled as ‘1’, ‘2’, ‘3’ in this example). In contrast, HCP does not have such symmetry. When one moves in the locally-defined grid axis, the grid field at the next layer is not passed (‘3’ does not pass the centre of the grid fields). It is easier to appreciate this 3D arrangement by using our visualization software ([www.fil.ion.ucl.ac.uk/Maguire/grid3D_gui](http://www.fil.ion.ucl.ac.uk/Maguire/grid3D_gui)).


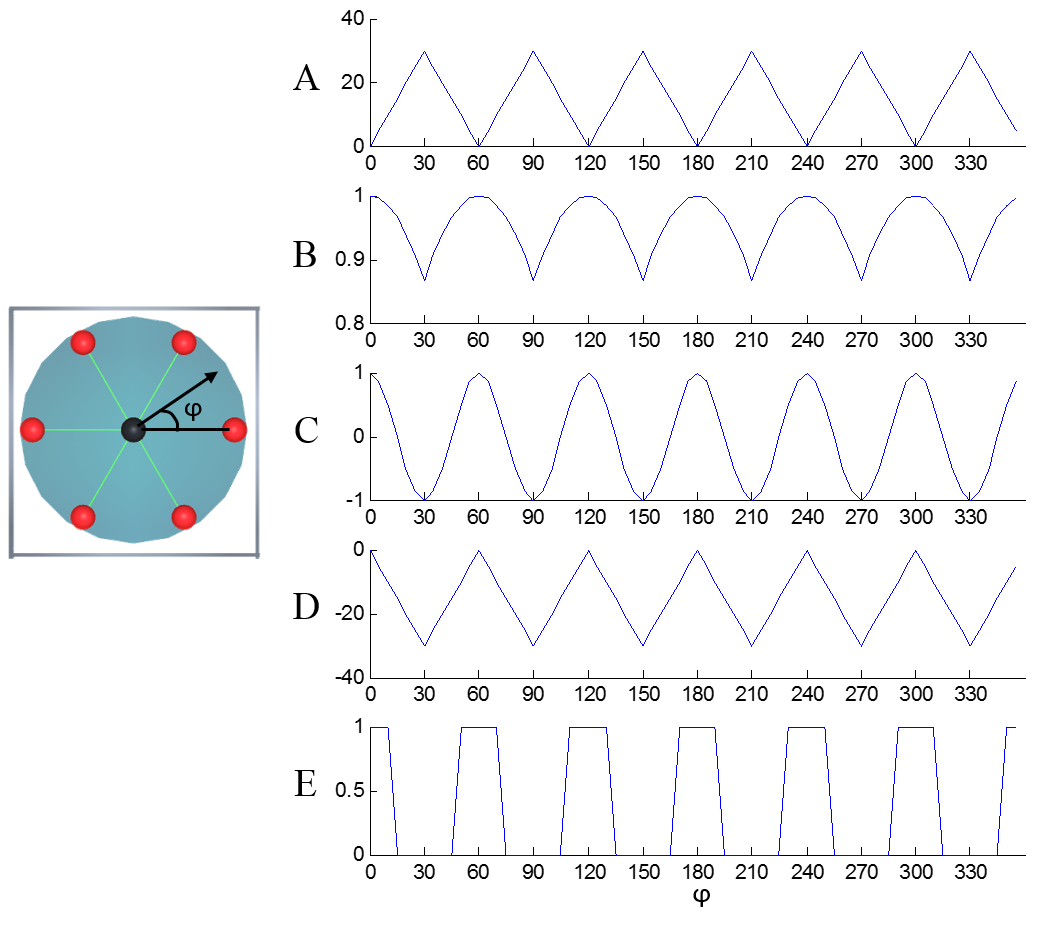


**Supplementary Fig. 2**. Possible relationships between grid alignment and the fMRI signal.

**A.** An angle between the movement direction (black arrow) and the nearest grid axis (green lines). **B.** A cosine of the nearest angle. **C.** A sinusoidal signal with a periodicity of 60 degrees. **D.** A linear signal (the negative of the nearest angle). **E.** A binary signal.


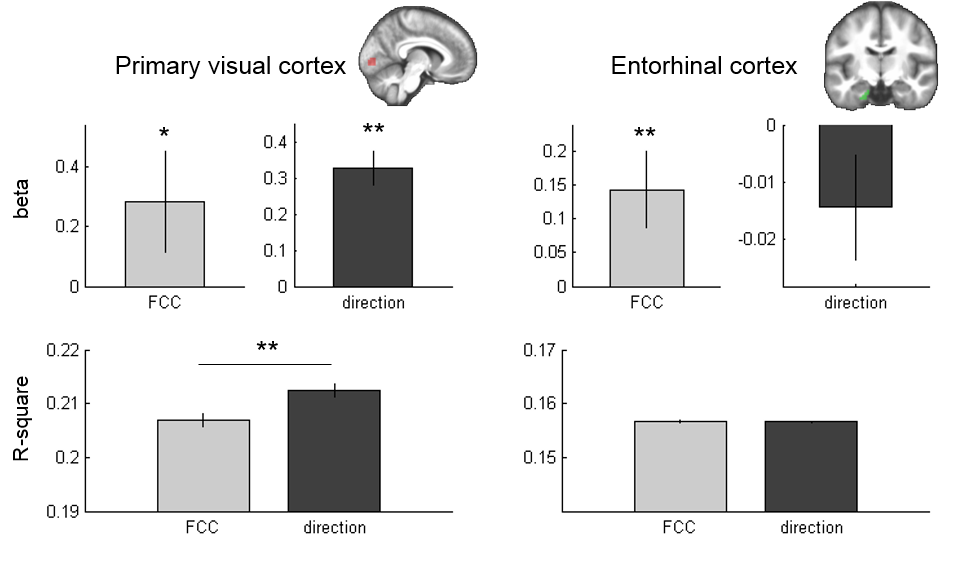


**Supplementary Fig. 3**. Comparison of primary visual cortex and the left entorhinal cortex.

For ease of comparison, on the left we reprise the entorhinal cortex result from the main text. The FCC grid model and the direction model were tested in primary visual cortex and the left entorhinal cortex. In the visual cortex, both the FCC and direction encoding models were significant. However, the FCC model fitted better than the direction encoding model (larger R-square). In contrast, only the FCC model was significant in the entorhinal cortex. * p<0.05, **p<0.01.

**Supplementary Text 1**. Comparison of numerical estimation and quadrature filter approaches in simulated 2D grid signals.

**Introduction**

In this simulation, we generated synthetic 2D grid signals to test whether our numerical grid orientation estimation method can detect such signals. We compared how well the numerical estimation methods with different sampling resolutions and a previous analytic approach (the quadrature filter, e.g. Doeller et al. 2010) detected the grid signal in the presence of noise.

**Methods**

We first created two virtual direction trajectories (approximating two scanning sessions of 10 minutes length each) where the range of movement (θ) was restricted to [-60°, 60°], similar to our empirical data (see Supplementary Fig. 4 below). Next, we computed the grid alignment score vector by assigning random grid orientations (ϕ) for 30 virtual participants using the formula, cos (6* (θ – ϕ)). This hypothetical grid cell activity was convolved with a canonical hemodynamic response function and then Gaussian noise of varying size (SNR=1, 0.1, 0.01) was added.

In the numerical estimation method, we iteratively fitted the hypothetical grid signals when different grid orientations were assumed (either 15°, 7.5° or 1° resolution) and selected the grid orientation which had the best fit to the actual data in each scanning session (see details in the main text). We tested whether the grid model selected in the first session resulted in a positive regression coefficient in the second session and vice versa (a one-sided t-test). For the quadrature filter approach, we estimated the grid orientation (ϕ) using the regression coefficient of cos(6*θ) and sin (6*θ) regressors, ϕ=arctan(b1/b2) for each scanning session. We tested whether a new cosine regressor with the grid orientation estimated from the first session resulted in a positive regression coefficient in the second session and vice versa.

**Results**

Supplementary Fig. 5 below shows group mean grid scores (beta) and R-square values for varying SNR. All numerical estimation methods and quadrature filter approaches could reliably detect the grid signal when SNR was high (Supplementary Fig. 5A, 15°, t(29)=33.5, p<0.001; 7.5°, t(29)=55.0, p<0.001; 1°, t(29)=97.3, p<0.001, quadrature, t(29)=98.1, p<0.001) or middle (Supplementary Fig. 5B, 15°, t(29)=5.3, p<0.001; 7.5°, t(29)=4.9, p<0.001; 1°, t(29)=5.0, p<0.001, quadrature, t(29)=5.1, p<0.001). None of the methods could detect the grid signal if the SNR was low (Supplementary Fig. 5C, 15°, t(29)=0.0, p=0.5; 7.5°, t(29)=-0.2, p=0.6; 1°, t(29)=-0.2, p=0.6, quadrature, t(29)=-0.2, p=0.6).

When SNR was high, the quadrature filter approach and numerical method with fine sampling resolution (1°, which virtually converges to an analytic solution) was better at detecting a grid signal compared to the numerical method with coarser sampling resolution (7.5°, 15°), in terms of a higher grid score and R-square (Supplementary Fig. 5A, grid score, F(3,87)=18.8, p<0.001; R-square, F(3,87)=27.9, p<0.001). This was due to discretized estimates of the grid orientation when coarser sampling resolution was used.

The numerical methods with varying sampling resolution and the quadrature filter approach showed similar grid scores and R-square values when SNR was middle (Supplementary Fig. 5B, grid score, F(3,87)=1.3, p=0.3; R-square, F(3,87)=1.0, p=0.4) or low (Supplementary Fig. 5C, grid score, F(3,87)=0.7, p=0.6; R-square, F(3,87)=2.2, p=0.1).

**Discussion**

Both analytic methods and numerical methods could detect underlying grid signals if the noise level was not too high. The analytic method and a numerical method with fine sampling resolution performed better than a numerical method with coarse sampling resolution if SNR was high. Real fMRI data is often noisy (e.g. the adjusted R-square value of our actual data in the entorhinal cortex was only 0.15) and the sampling resolution of the numerical method might have a negligible effect on detecting grid signal. Indeed, FCC grid scores in our actual fMRI data were comparable when we estimated the grid orientation with either 15° or 7.5° resolution.


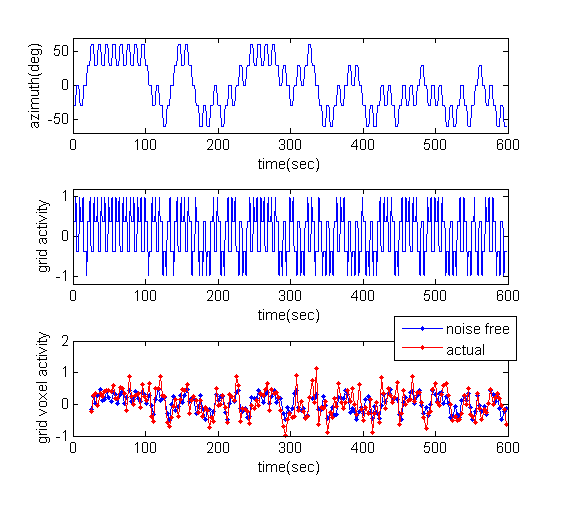


**Supplementary Fig. 4.** Simulated grid cell activity. Top panel, virtual movement trajectory of a participant. Middle panel, hypothetical grid cell activity of one participant whose grid orientation (ϕ) was 41°. Bottom panel, hypothetical grid cell activity was convolved with a hemodynamic response function, resulting in grid voxel activity. Before (“noise free”) and after (“actual”) the addition of Gaussian noise (SNR=1).


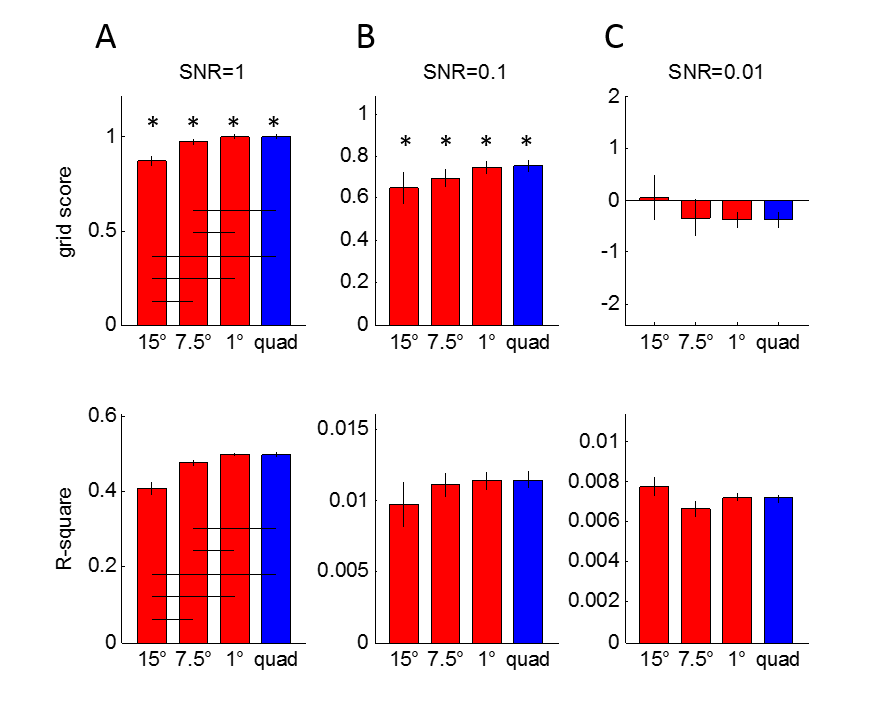


**Supplementary Fig. 5.** Comparison of the numerical estimation and quadrature filter approaches in 2D simulated data. When SNR was relatively high (**A** and **B**), both numerical and filter approaches correctly revealed the grid signal (positive grid score). When SNR was low (**C**), neither of the methods recovered the grid signal. Quadrature filter approaches or the numerical estimation with fine resolution (1°) were more sensitive to detecting grid-like signals than numerical estimation methods with coarse sampling resolutions (15°, 7.5°), only when SNR was high (**A**). All methods yielded similar results when SNR was middle or low (**B** and **C**). *p<0.05. Black horizontal bars indicate a significant difference between the conditions (post-hoc pairwise t-tests). Error bars are standard error adjusted for a within-subjects design.

**Supplementary Text 2**. Comparison of the FCC and HCP models in simulated 3D grid signals.

**Introduction**

In this simulation, we generated synthetic 3D grid signals to test whether our numerical grid orientation estimation method can correctly identify the FCC and HCP models. We already discussed the challenge of detecting the HCP model due to its absence of global grid axes (Methods section in the main manuscript), and for the purpose of comparing the two models, we assumed here that the HCP model is modulated by locally defined grid axes. The goal of this simulation was to investigate whether the two models could be distinguished despite their similarities and the presence of noise when limited movement directions were sampled.

**Methods**

We used virtual 3D trajectories equivalent to those used in the actual experiment (from -60 to 60 degrees vertically and horizontally, ~240 TRs for each scanning session) (Supplementary Fig. 6A). We assigned random grid orientations for 30 virtual participants (0, 7.5, 15, 22.5, …,120 degrees) and created a direction-modulated grid signal that followed either the FCC or HCP model (=cosine of the angle between the movement directions and the nearest grid axis) (Supplementary Fig. 6B). We reiterate that the exact form of the direction-modulated signal is unknown in the HCP arrangement because of the lack of a global grid axis, and here we assumed that the HCP signal would be dependent on the local grid axes. A canonical hemodynamic response function was applied and Gaussian noise was added to the hypothetical grid signal (SNR=1 or 0.05).

We estimated the grid orientation of each session by iteratively fitting the hypothetical grid signals, separately for the FCC or HCP models with 15° resolution. We tested whether a regression coefficient (grid score) for the selected grid model was positive at the group level (a one-sided t-test). We compared the grid score and R square between the FCC and HCP models when the true data were generated with either the FCC or HCP models.

**Results**

When the SNR was high (SNR=1), the FCC model showed a higher grid score and larger R squared than the HCP model when the true model was the FCC (Supplementary Fig. 6C), and vice versa when the true model was the HCP (Supplementary Fig. 6D). This means that the two models were correctly and unequivocally distinguished. Of note, grid scores were significantly positive for both FCC and HCP models regardless of whether the true model was FCC or HCP. This was due to the similarity between the FCC and HCP alignments (e.g. 9 out of 12 neighbouring fields were at the same 3D locations for the two alignments, Figure 2 in the manuscript).

When the SNR was low (0.05), we repeated the simulation 100 times by changing the random number generator setting (“rng” function in MATLAB) to avoid the risk of reporting an extreme case in this low SNR regime. Here, we describe the cases when the true model was FCC (the results were similar when the true model was HCP). In the majority of simulations (87 out of 100, an example is shown in Supplementary Fig. 6E), both FCC and HCP models showed positive grid scores. Unlike the high SNR case where a true model showed higher grid scores and R square, grid scores were comparable for the two models in 76 cases and only 11 cases showed a higher grid score for the correct FCC model. R-squares were also similar in most cases. This means that the two models could not be distinguished. Of note, there were 8 cases where only the FCC model was significantly positive, and the grid score of the HCP model was not (Supplementary Fig. 6F). These simulation cases resonated with the actual fMRI data we observed (only the FCC model was significant in the left entorhinal cortex). In 2 cases, only the wrong model (HCP) was significant.

**Discussion**

Based on the simulation results, we predict that if the true grid signal is large, FCC and HCP models would be correctly identified using our numerical estimation methods even when the sampled direction are limited to 120 degrees. When the SNR is low, our analysis will pick up evidence of a 3D grid signal, but it will not permit identification of which model was the correct one.


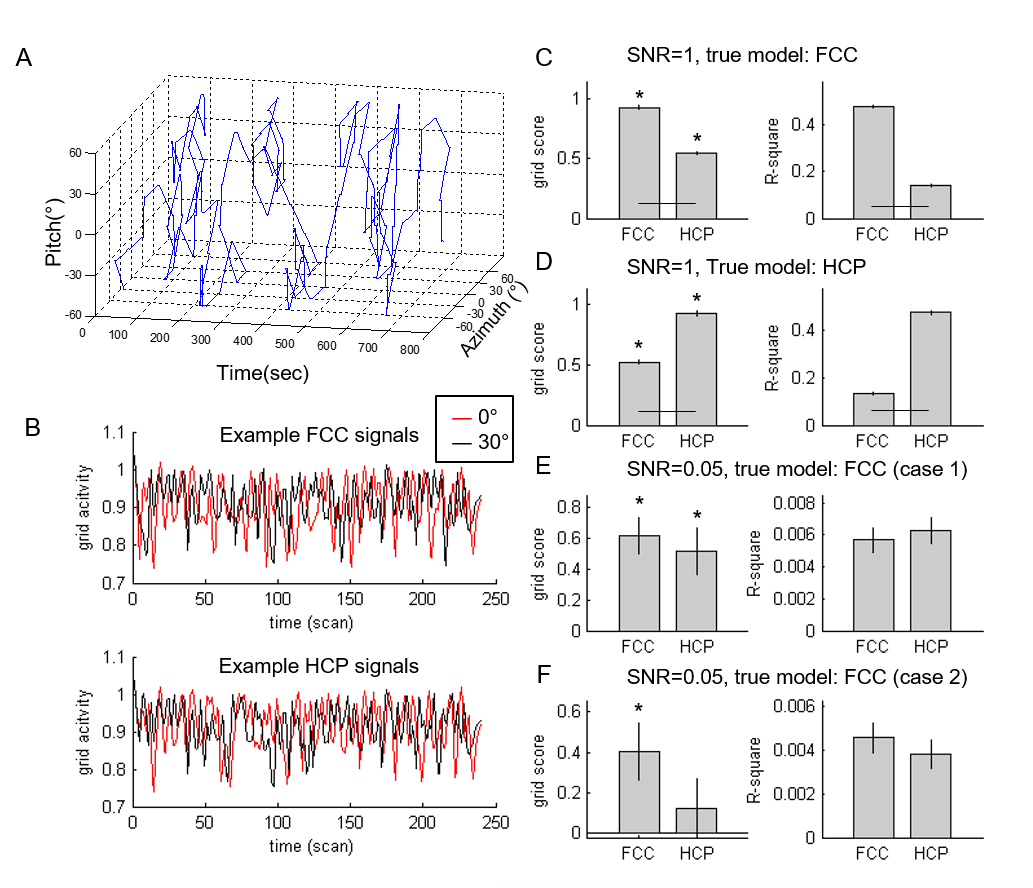


**Supplementary Fig. 6.** Simulation of the FCC and HCP models. **A.** A virtual 3D trajectory. Movement directions were limited to -60° to 60° vertically (pitch) and horizontally (azimuth). **B. A** hypothetical grid voxel responses when grid cells followed either the FCC (the top panel) or the HCP (the bottom panel) model. A grid cell’s activity is dependent on the grid orientation and movement direction. Two examples grid signals are shown when the orientation was 0° (red) or 30° (black). **C-F.** Simulation results when different SNR and different grid models were used. Thirty virtual participants were used. When the SNR was high, the FCC grid signal was correctly identified as the FCC model (higher grid score and R square for the FCC model compared to the HCP model) (**C.**), and the HCP grid signal was correctly identified as HCP model (higher grid score and R square for the HCP model) (**D.**). When the SNR was low, grid scores and R square were not significantly different for the FCC and HCP model fits (**E,F.**). In some cases, only the correct model showed a significantly positive grid score (**F.**). *p<0.05.
